# Determination of Methyldopa and Paracetamol in Pharmaceutical Samples by a Low Cost *Genipa americana* L. Polyphenol Oxidase Based Biosensor

**DOI:** 10.15171/apb.2019.049

**Published:** 2019-08-01

**Authors:** Rafael Souza Antunes, Douglas Vieira Thomaz, Luane Ferreira Garcia, Eric de Souza Gil, Vernon Sydwill Sommerset, Flavio Marques Lopes

**Affiliations:** ^1^Faculdade de Farmácia, Universidade Federal do Goiás (UFG), rua 221 esquina com a 5ª avenida s/n, Setor Universitário, Goiânia-GO, Brasil.; ^2^Department of Chemistry, Faculty of Applied Sciences, Cape Peninsula University of Technology, Bellville 7535, South Africa.

**Keywords:** Jenipapo (*Genipa americana* L.), Biosensor, Methyldopa, Paracetamol

## Abstract

***Purpose:*** Jenipapo fruit (*Genipa americana* L) is a natural source of polyphenol oxidases (PPOs) whose potential in pharmaceutical analysis is noteworthy. Henceforth, this work reports the electrochemical study of a low-cost PPO-based biosensor produced from the crude extract of Jenipapo fruits and accounts a practical approach to employ this biosensor in the determination of methyldopa and paracetamol in pharmaceutical samples.

***Methods:*** In order to investigate the electrochemical properties of the biosensor, theoretical and practical approaches were employed, and both samples and the biosensor were analyzed through electrochemical impedance spectroscopy (EIS) and voltammetric techniques, namely: differential pulse voltammetry (DPV) and cyclic voltammetry (CV).

***Results:*** showcased that the biosensor presented good analytical features, as well as low detection limits (8 μmol L-1 for methyldopa and 5 μmol L-1 for paracetamol). The relative standard deviation was less than 5% mid-assay.

***Conclusion:*** The use of this biosensor is a reliable, low cost and useful alternative in the pharmaceutic determination of phenolic drugs (e.g. methyldopa and paracetamol).

## Introduction

Biosensing technology is a growing field in science whose potential is being gradually explored in analytical chemistry. The biological element in biosensors allows higher selectivity through enzymatic mechanisms, which in turn, increases sensitivity through the magnification of the generated chemical signal. In this context, enzymes possess optimal bio-catalytic proprieties which can be used in biosensor development, and provide unmatched fast analysis, reproducibility and low-cost detection.^1,2^

Amongst the enzymes employed in biosensor technologies, polyphenol oxidases (PPOs) are noteworthy due to their universal distribution in vegetal tissues and numerous applications in literature concerning their biosensing applicability. PPOs are readily available in crude vegetal extracts, and this feature allows fast and simple extraction, as well as the use of low-cost solvents such as water. This group of enzymes is responsible for the oxidation of phenols to quinones, and the reduction of quinones through potential sweeping generates cathodic faradaic signals, which can be transduced into voltammetric data.^1,3,4^

Concerning the immobilization of enzymes in biosensor production, carbon-based matrixes such as carbon paste (CP) are the most employed in literature, since they allow clear faradaic current detection without elevated capacitive signal generation. Moreover, CP is easily modified through chemisorption, adsorption or covalent binding of biological macromolecules, which increase signal detection and henceforth, assay sensibility.^5,6^

Literature accounts that PPO-based biosensors are remarkable for pharmaceutical analysis. In this context PPO rich materials such as jenipapo (*Genipa americana* L.) might be useful in biosensing technologies. Jenipapo is a Brazilian Cerrado fruit whose abundance of phenolic compounds turns it into a valuable ingredient in folk medicine.^7,8^ Nonetheless, Brazilian Cerrado fruits have been used by our group to produce biosensors to evaluate pharmaceutical samples and phenolic contaminants in effluents from textile industries, which further strengthens the appeal of this vegetal towards biosensor development.^9-11^

The use of drugs whose structures contemplate phenolic moieties is widespread in medicine.^12,13^ Amongst these compounds, methyldopa and paracetamol are distinctive due to their status as two of the most consumed drugs in the world. Although both compounds possess similar structures, their pharmacodynamic properties are distinct, as methyldopa is a α2 inhibitor, while paracetamol is a non-steroidal anti-inflammatory drug.^2,12,13^

Although their determination in pharmaceutical samples is already well reported, most methods are nonetheless expensive, and comprise mostly of high performance liquid chromatography techniques coupled to UV-Visible spectrophotometry (HPLC-UV/Vis), which leads to the use of high amounts of solvents. This drawback, as well as the limited selectivity possessed by colorimetric techniques can be outmatched by electrochemical tools, which may be moreover, associated to PPO based biosensors.^14-16^

Therefore, this work showcases the study of the electrochemical features of a previously developed jenipapo-PPO-based biosensor (JeEE@CP)^9^ and investigate its applicability to determine methyldopa and paracetamol in pharmaceutical samples.

## Material and Methods

### Reagents and Solutions

Potassium ferrocyanide and KCl were purchased from Vetec Química Fina Ltda. (Rio de Janeiro, Brazil) and diluted in purified water (conductivity ≤0.1 µS.cm^-1^) obtained from Milli-Q purification system Millipore S/A (Molsheim, França) in order to reach a final concentration of 0.001 mol L^-1^. Thereafter, KCl was added to this solution up to a concentration of 0.1 mol L^-1^.

Methyldopa and paracetamol standards were purchased from Sigma-Aldrich (St. Louis, MO, EUA) and their stock solutions were prepared to render 100 µM solutions. Methyldopa and paracetamol commercial tablets were donated by the pharmacy of the Faculty of Pharmacy of the Federal University of Goiás (Goiás, Brazil).

### Biosensor production

The biosensor used in this study was produced according to a previously described protocol, which was optimized by our group in a previous outreach,^9^ using a crude vegetal extract obtained from Jenipapo fruits. The vegetal material was collected from a single plant located in the district of Anápolis-GO, Brazil in January 2017. Geographic coordinates: 16°19’36” S 48°57’10” W. A total of 10 fruits were collected, rinsed with water and stored in polyethylene containers at 4°C until analysis.

Crude vegetal extract was prepared by milling 30 g of fruits for 2 minutes in a food processor (Britania, Brazil) and adding 100 mL phosphate buffer solution (PBS) 0.05 mol L^-1^ (pH 6.0). The solution was homogenized and filtered with sieving cloth, leading to a crude vegetal extract at 30% (Jenipapo enzymatic extract, JeEE) 0.01 mol L^-1^ (pH 6.0). All procedures were conducted at room temperature (20°C ± 2°C).

To produce the biosensor, CP was firstly prepared using graphite powder and mineral oil purchased from Sigma-Aldrich (St. Louis, MO, USA). Enzyme immobilization from JeEE was performed by physical adsorption on CP. Approximately, 100 μL of the enzyme extract was added directly into 100 mg of graphite powder and then homogenized and dried at room temperature (20°C ± 2°C). The mineral oil (30 mg) was then added and the entire mixture was further homogenized.

All voltammetric procedures conducted with the JeEE@CP biosensor were optimized in a previous report by our group, and the best pH for analysis was of 7.0.^9^

### Electrochemical assays

Electrochemical impedance spectroscopy (EIS) and voltammetric measurements were performed using a potentiostat/galvanostat PGSTAT^®^ model 204 with FRA32M module (Metrohm Autolab) integrated with NOVA 2.1^®^ software. All measurements were performed in a 1 mL one-compartment electrochemical cell coupled to a three-electrode system consisting of the biosensor herein employed, Pt wire and Ag/AgCl/KCl_sat_ (both purchased from Lab solutions, São Paulo, Brazil). The electrodes cited above represent: working, counter and reference electrode, respectively.

EIS measurements were conducted in a solution containing 1 mmol L^-1^ potassium ferrocyanide and 0.1 mol L^-1^ KCl over a frequency ranging from 0.01 Hz to 100 kHz at selected potentials for all tested electrodes.

The experimental conditions for cyclic voltammetry (CV) were: scan rate (υ) of either: 12.5; 25; 50; 100; 250 or 500 mV s^-1^, and scan range of -0.5 to 1.0 V. Differential pulse voltammetry (DPV) conditions were: pulse amplitude 50 mV, pulse width 0.5 s and scan rate 10 mV s^-1^. All voltammetric assays were performed either in 0.1 M PBS pH 7.0, or in 1 mmol L^-1^ potassium ferrocyanide / 0.1 mol L^-1^ KCl solution. Experiments were moreover conducted at room temperature (21ºC ± 2ºC) in triplicates *(n = 3*) to ensure reproducibility.

Plots of the voltammetric curves for final presentation in this study were drawn using Origin Pro 8^®^ software (Northampton, MA, USA).

### Calibration curve, limit of detection (LoD), standard recovery and pharmaceutical sample analysis

All analytical parameters were calculated according to the Brazilian National Agency of Sanitary Vigilance.^17^ Calibration curves were prepared using DPV assays in a linear range from 10 to 310 µmol L^-1^ of either methyldopa or paracetamol. The concentrations herein used were reached upon dilution of stock solutions in PBS pH 7.0. The standard recovery was calculated from the difference between the detected amount of the analyte, and the amount of added standard (50, 100 and 200 µmol L^-1^).

The pharmaceutical samples herein used contained 250 and 500 mg of methyldopa and paracetamol, respectively. The samples were prepared according to the procedures described in the 5^th^ edition of the Brazilian pharmacopoeia,^18^ in which 10 tablets of each medication were grounded separately, and an amount enough to render 1 mmol L^-1^ solution of each drug was solubilized and filtrated. The prepared 1 mmol L^-1^ solution was further diluted to 100 µmol L^-1^. UV-Visible spectrophotometry, which is described in the Brazilian Pharmacopeia to assess methyldopa and paracetamol, was herein used to compare results, and readings were conducted at 257 nm and ambient temperature.

### Statistical analysis

All statistical analysis was conducted in BioEstat^®^ software, version 5.3. The data was evaluated using Tukey test, and statistical significance was considered to *P* < 0.05.

## Results and Discussion

### CV and EIS assays

**Figure 1:**
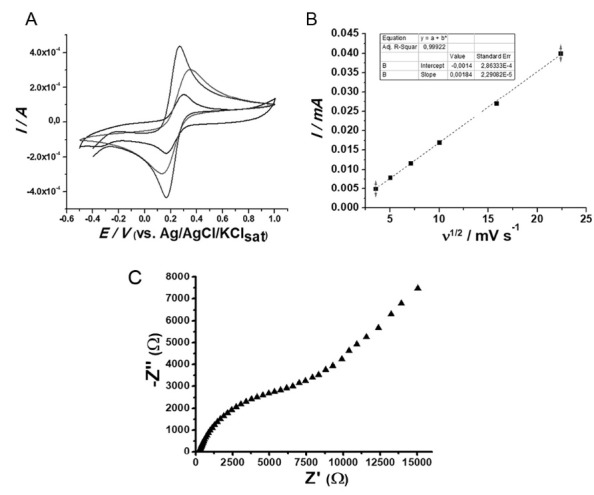
**(A)** Ciclic voltammograms taken from ferrocyanide solution using different electrodes, namely: JeEE@CP biosensor (intermediary amplitude); GCE (smallest amplitude), and CP electrode (highest amplitude). All analysis were performed at υ of 100 mV s^-1^ in 1 mmol L^-1^ potassium ferrocyanide/0.1 mol L^-1^ KCl. **(B)** Plot of peak current (*I*) vs scan rate square root (υ^1/2^) of: JeEE@CP. **Insert.** Data of the linear fit. All analysis carried out in 1 mmol L^-1^ potassium ferrocyanide/0.1 mol L^-1^ KCl. **(C)** EIS nyquist plot of JeEE@CP (▲). All analysis carried out in 1 mmol L^-1^ potassium ferrocyanide/0.1 mol L^-1^.

In order to preliminarily investigate electrodic response in absence of the biocatalytic effect, the biosensor was subjected to CV analysis in a probe which exhibits diffusion-controlled electrochemical process (*i.e.* potassium ferrocyanide/KCl solution) and does not undergo redox reactions in the presence of PPOs. Results are displayed in Figure 1A.

Results indicate that the jenipapo-PPO modification decrease ferrocyanide probe faradaic current amplitude in relation to other electrodes, what suggests that the biological component (*i.e.* PPO) hinder electric transfer. This finding is nonetheless in accordance to literature, since plant extract-based biosensor applicability is only feasible due to the catalytic properties of the biological component (which thence magnifies signal).^10,11^ Given that the ferrocyanide probe does not undergo catalysis through PPO and the biological component reduces electric current output, its signal was therefore expected to be the smallest.

Considering that electrode surface area also influences signal output, JeEE@CP anodic peak currents were taken from voltammograms recorded at different scan rates. Results are displayed in Figure 1B.

The plot presented linear tendency between data from faradaic current peaks and square root of scan rate’s (Figure 1B), which is in accordance with literature and experimental conditions, since data was taken in potassium ferrocyanide, which is a probe known for its diffusion controlled electrochemical process.^16,19,20^ Moreover, electrode surface area was determined by the Randles-Sevcik equation (1), using a 1 mmol L^-1^ potassium ferrocyanide and 0.1 mol L^-1^ KCl solution as a reversible one-electron diffusion controlled redox system.

*I*_pa_ *= 2.69 10* ^5^ *A n* ^3/2^ *D* ^1/2^ *c υ* ^1/2^ (1)

Wherein: *I*_pa_ is the anodic peak current, A is electrode area in cm^2^, *n* is the number of transferred electrons, *D* is the diffusion coefficient, which was estimated to be 7.09·10^−6^ cm^2^ s^−1^ (Konopka and Mcduffie, 1970), *c* is the concentration of potassium ferrocyanide/KCl in mol L^-1^, and υ is scan rate in V s^-1^. The values of *I*_pa_/υ^1/2^ were obtained from the slopes of the curves displayed in Figure 1B. The surface area of the biosensor was estimated to be 9.67 mm^2^.

Literature states that electrode area and the nature of the coating component are nonetheless important when signal gathering is concerned.^16,21^ However, the electroactive area may be hindered by biological components such as PPO, what promotes smaller anodic signals even with larger surface areas. Therefore, a biosensor is only reliable for its specific analyte (e.g. PPO biosensors for phenolic analytes), what is supported by the fact that the analytical increment of biosensors is promoted mainly by the biological activity of the macromolecule used in its construction. Given that enzymes are substrate-specific, the results taken in ferrocyanide probe are in consonance to literature, since this electrochemical probe does not undergo biological catalysis.^1,4,8,10^

Regarding the high surface area of the biosensor, scanning electron microscopy results from previous outreaches of our group showcased that JeEE coated CP electrodes present irregular surface,^9^ which further explain the findings herein reported.

EIS analysis was also conducted since circuit parameters are essential to better understand electrode behavior. Results are displayed in Figure 1C, and all data concerning the Randles equivalent circuit of JeEE@CP was gathered and displayed in Table 1. Where *R*_s_ is the uncompensated Ohmic resistance (Ω); *R*_ct_ represents the resistance associated to charge transfer (Ω); *C* and *n* are respectively the pseudo-capacitance (µF) and frequency independent taken from the constant phase element, which describes the imperfect capacitive behavior of the double-layer.

**Table 1:** Randles equivalent circuit elements for JeEE@CP

**Electrode**	**Circuit elements**	**Circuit elements**
JeEE@CP	*R*_s_	209
	*R*_ct_	7945
	*n*	0.655
	*C*	5.930

Results showcased that JeEE@CP biosensor exhibited average *n* values, these values range from 0 to 1 and are descriptors for either the resistive or capacitive behavior exhibited by the electrode.^19^ Albeit solid electrodes with highly conductive surfaces tend to present values close to 1, JeEE@CP presented 0.655 possibly due to the organic portion of the matrix, what hinders double layer formation. Another noteworthy finding is that *R*_ct_ value for JeEE@CP is higher than the ones reported in literature for similar CP based electrodes, which may be justified by the non-electro-activity displayed by the biological fraction, thus increasing resistance to electron transfer.^5,6^

Furthermore, the biosensor was employed to assess methyldopa and paracetamol in pharmaceutical samples.

### Analytical curve and drug determination

**Figure 2:**
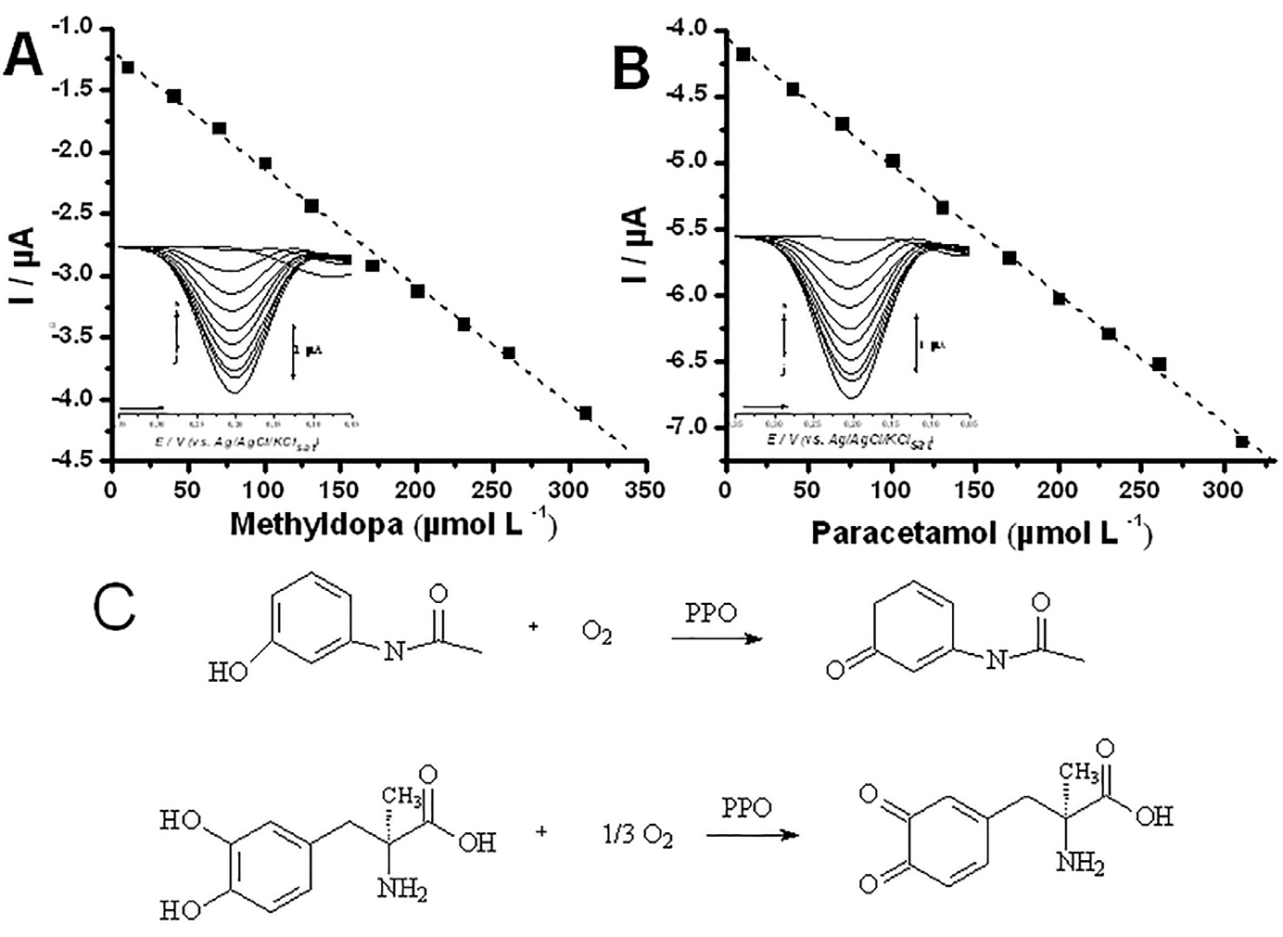
Calibration curves obtained for: **(A)** methyldopa and **(B)** paracetamol. **Inserts.** DPV assays of: **(A)** methyldopa and **(B)** paracetamol. All experiments conducted in PBS 0.1 mol L^-1^, pH 7.0 at JeEE@CP. **(C)** PPO mediated oxidation of methyldopa and paracetamol.

In order to confirm whether the detection of both methyldopa and paracetamol presented linearity, analytical curves were constructed and evaluated. Results demonstrated that both analytes presented good linearity through DPV assays conducted using JeEE@CP biosensor (10 to 310 µmol L^-1^ for both analytes, and r^2^ of 0.9983 and 0.9992 for methyldopa and paracetamol, respectively). Peak amplitude varied from 1.308 to 4.102 μA for methyldopa and 4.181 to 7.097 μA for paracetamol, while presenting LoDs of 8 μmol L^-1^ and 5 μmol L^-1^, respectively (Figure 2A-B).

Previous reports from our group demonstrated that JeEE@CP biosensor is providing more sensitive results than unmodified electrodes when the detection of phenolic compounds is concerned.^9^ Since this enhancement in sensitivity is stated to be a direct effect of PPOs present in JeEE, we therefore proposed a mechanism involving enzymatic oxidation of both methyldopa and paracetamol phenolic moieties.

The proposed mechanism is well stated in literature to phenolic compounds,^9-11^ and is demonstrated in Figure 2C, where the formation of the quinones therein shown leads to the enhancement of the faradaic signal gathered in the cathodic scan of DPV (reduction).

Furthermore, the analytical performance of the JeEE@CP biosensor towards the assessment of both methyldopa and paracetamol was compared to other methods described in literature. Findings are displayed in Table 2.

**Table 2:** Comparison between analytical performance of JeEE@CP biosensor and other methods in literature

**Analyte**	**Electrode**	**Linearity (µmol L** ^-1^ **)**	**Limit of Detection (µmol L** ^-1^ **)**	**References**
Methyldopa	CA/BMI-N(Tf)_2_	34.8–370.3	5.5	^22^
	3,4’AAGCPE	10–45	9	^23^
	JeEE@CP	10–310	8	Present work.
Paracetamol	Zucchini-CPE	120–2500	69	^24^
	Avocado-CPE	120–5800	88	^25^
	BH-CPE	10–250	1.6	^26^
	Cu^2+^Y/ZMCPE	0.25–900	0.1	^27^
	JeEE@CP	10–310	5	Present work.

As demonstrated in Table 2, the method herein proposed is comparable to other methods reported in literature, and in some cases, even surpasses their analytical performance, therefore further demonstrating the usefulness of the JeEE@CP biosensor towards methyldopa and paracetamol analysis.

### Standard recovery and pharmaceutical analysis

In order to evaluate the precision and accuracy of the JeEE@CP biosensor, a standard recovery test was conducted. Results are displayed in Table 3.

**Table 3:** Methyldopa and paracetamol standard recovery test results using JeEE@CP biosensor (n = 3)

**Analyte**	**Added (µmol L** ^-1^ **)**	**Expected (µmol L** ^-1^ **)**	**Recovery (µmol L** ^-1^ )*	Standard Error (%)**	**Recovery (%)**
Methyldopa (100 µmol L^-1^)	0	100	99.25 ± 0.22	0.75	99.25
	50	150	144.92 ± 0.19	3.38	96.61
	100	200	194.99 ± 0.42	2.50	97.49
	200	300	305.73 ± 0.83	1.91	101.91
Paracetamol (100 µmol L^-1^)	0	100	100.33 ± 0.46	0.33	100.33
	50	150	148.02 ± 0.32	1.32	98.68
	100	200	200.89 ± 0.98	0.44	100.44
	200	300	295.97 ± 0.11	1.34	98.65

*Amount recovered through DPV assay. **Standard error between the concentration expected and the one found.

The amounts of analytes recovered through the tests were around 0.33% and 3.38%, which is below the limit of 5%. Therefore, the DPV method of analysis using the JeEE@CP biosensor presents precision and accuracy in accordance to these analytical criteria, and was henceforth tested to determine methyldopa and paracetamol in pharmaceutical samples.

Pharmaceutical samples of methyldopa and paracetamol were assessed using the UV-Vis spectroscopy and JeEE@CP/DPV method. Tukey’s test was henceforth used to compare results and the observation are results are displayed in Table 4.

**Table 4:** Results obtained from methyldopa and paracetamol tablets using UV-Vis spectrophotometric method and JeEE@CP biosensor voltammetric method (JeEE@CP/DPV) (n = 3)

**Drugs**	**Informed amount (mg)**	**UV-Vis (mg)**	**JeEE@CP/DPV (mg)**	Recovery (%)*	Recovery (%)**	Standard Error (%)***
Methyldopa^a^	250	261.79 ± 1.37	257.98 ± 0.92	104.71	103.19	1.52
Paracetamol^b^	500	502.93 ± 1.13	493.09 ± 0.31	100.58	98.61	1.96

^a^ *P* = 0.0103; ^b^*P* = 0.0267 *Amount recovered from UV-Vis method. **Amount recovered from JeEE@CP/DPV method. ***Standard error between the amounts found from UV-Vis assay and JeEE@CP/DPV assay in comparison to the informed amount in pharmaceutical tablets.

Results demonstrated that no statistically significant difference was found between the assayed values of UV-Vis spectrophotometric method and the JeEE@CP/DPV method. The recovered amount of each analyte in pharmaceutical samples was 98.61% for methyldopa and 104.71% for paracetamol, which is in accordance to pharmaceutical compendia.^17,18^ These findings further indicate that the method herein proposed is a low cost and analytically viable tool to determine methyldopa and paracetamol in pharmaceutical samples.

## Conclusion

The voltammetric method using a jenipapo based biosensor presented good sensitivity towards biosensor detection as well as good detection limits (8 μmol L^-1^ for methyldopa and 5 μmol L^-1^ for paracetamol). The results obtained for the JeEE@CP biosensor also showed a linear range from 10–310 μmol L^-1^ for both methyldopa and paracetemol analysis. The relative standard deviation was less than 5% mid-assay, which configures the developed biosensor as a reproductive, low cost and useful alternative to assess these drugs in pharmaceutical samples.

## Ethical Issues

Not applicable.

## Conflict of Interest

Authors declare that there is no conflict of interest.

## Acknowledgments

The authors are grateful to CNPq (Grants 456211 / 2014-4), the Foundation for Research Support of the State of Goiás (FAPEG) and the Coordination for the Improvement of Higher Education Personnel (CAPES) (AUXPE 1665/2016) for financial support.
